# Efficacy of PCV2 Vaccination Under Natural Conditions: A Longitudinal Study Using PCR and Virus Isolation

**DOI:** 10.3390/vetsci12060575

**Published:** 2025-06-11

**Authors:** Eugene Mazimpaka, Rissar Siringo Ringo, Tasuku Hirooka, Tamaki Okabayashi

**Affiliations:** 1 Graduate School of Medicine and Veterinary Medicine, University of Miyazaki, Miyazaki 889-2192, Japan; mazimpaka_eugene@med.miyazaki-u.ac.jp (E.M.); rissar_siringo_ringo@med.miyazaki-u.ac.jp (R.S.R.); 2 Department of Veterinary Science, Faculty of Agriculture, University of Miyazaki, Miyazaki 889-2192, Japan; gf19022@student.miyazaki-u.ac.jp; 3 Center for Animal Disease Control, University of Miyazaki, Miyazaki 889-2192, Japan

**Keywords:** porcine circovirus type 2, porcine reproductive and respiratory syndrome virus, co-infection, vaccine

## Abstract

Porcine circovirus type 2 (PCV2) is the main cause of porcine circovirus-associated disease (PCVAD). Despite the widespread use of anti-PCV2 vaccines, their efficacy varies, influenced by co-infection and evaluation methods. This study assessed the efficacy of Ingelvac CircoFLEX^®^ PCV2 vaccine under natural conditions. One hundred serum samples were collected from vaccinated and non-vaccinated piglets aged 21 to 173 days. PCR and antibody positivity rates did not show significant differences between the two groups, but PCV2 gene load at 91 days was significantly lower (*p* = 0.0095) in the vaccinated group. Anti-PCV2 antibody titers were also significantly lower in the vaccinated group at 91, 145, and 173 days (*p* < 0.0001). PCV2 was isolated from 50% of piglets in the non-vaccinated group (50%), compared with none (0%) in the vaccinated group, suggesting that PCV2 gene load in the non-vaccinated group did not correlate with viremia. Both groups were positive for antibodies to porcine reproductive and respiratory syndrome virus (PRRSV) at 63 days, prior to the surge in PCV2 gene load, suggesting PRRSV may enhance PCV2 replication. These findings highlight that while the vaccine reduced PCVAD damage, evaluation should incorporate methods such as virus isolation instead of relying solely on PCR.

## Simple Summary

Porcine circovirus type 2 (PCV2) causes porcine circovirus-associated disease. This study assessed the efficacy of the PCV2 vaccine (Ingelvac CircoFLEX^®^) under natural conditions, including age, evaluation methods, and co-infection in piglets on farms. One hundred serum samples were collected from vaccinated and unvaccinated piglets. PCR and antibody positivity rates were similar in both groups. These results suggest that qPCR detection of PCV2 genes in blood alone is insufficient to assess vaccine effectiveness, and additional methods, such as virus isolation, are needed. Porcine reproductive and respiratory syndrome virus (PRRSV) infection, observed at a low level on day 63 before PCV2 infection at 91 days, may be involved in PCV2 replication. This study found that vaccination against PCV2 efficiently reduced the negative effects of PCV2 replication and effectively controlled PCV2 infection under field conditions.

## 1. Introduction

Porcine circovirus type 2 (PCV2) is the smallest non-enveloped, single-stranded DNA virus belonging to the genus *Circovirus* of the family *Circoviridae*. PCV2 is one of the most economically relevant viruses for the swine industry, as it is the primary causative agent of porcine circovirus-associated disease (PCVAD), a multifactorial condition responsible for major losses in the swine industry [1,2]. The most common clinical manifestation of PCVAD diseases is post-weaning multisystemic wasting syndrome (PMWS), which was first described in Canada in 1991 [3]. Other clinical manifestations of PCVAD include porcine dermatitis and nephropathy syndrome (PDNS), porcine respiratory disease complex (PRDC), and reproductive failure. Vaccines against PCV2 have been shown to control disease manifestations and to improve production parameters [4,5,6], but the efficacy of these vaccines against PCV2 infection in the field has been reported to vary [7].

PCR is a useful method for detecting the PCV2 genome [8,9]. Although PCV2 vaccines were found to improve productivity in pigs with and without PMWS, high levels of PCV2 have been detected using PCR in vaccinated pigs [7,10]. Thus, PCR quantitation of the PCV2 genome does not indicate viremia, suggesting that isolation of PCV2 is necessary to evaluate its viremic status. Because PCV2 generally does not have a cytopathic effect on cultured cells [11,12]. The low growth efficiency of the virus has become a major obstacle to establishing diagnostic methods for PCV2 [13,14].

Although PCV2 is the primary cause of PCVAD, this virus is seldom the sole pathogen in pigs with PCVAD. Rather, many of these pigs are co-infected with porcine reproductive and respiratory syndrome virus (PRRSV), a single-stranded, enveloped RNA virus in the genus Arterivirus of the family *Arteriviridae* [15,16]. PRRSV is a causative agent of porcine reproductive and respiratory syndrome (PRRS), a condition closely related to PRDC [17]. Co-infection with PCV2 and PRRSV is common in pigs with clinical conditions contributing to a variety of polymicrobial disease syndromes, with rates of co-infection ranging from 42% to 85.4% in post-weaned pigs [18]. Furthermore, co-infection with PCV2 and PRRSV can result in more severe clinical signs as well as higher morbidity and mortality rates in pigs than infection with either virus alone [15].

The present study evaluated the efficacy of a PCV2 vaccine on piglets on a farm using quantitative PCR (qPCR) and virus isolation. This study also investigated the possibility of co-infection and its potential impact on PCV2 status.

## 2. Materials and Methods

### 2.1. Farm Condition

This study involved animals at a conventional pig farm located in Kumamoto, Japan. This farm was a two-site herd of all sows with all-in/all-out management and a 4-week batch farrowing system. Sows were routinely vaccinated against PCV2 but not PRRSV. Piglets were weaned at 21 days of age and moved to fattening units at 70 days. All pigs were checked for respiratory signs, inappetence, lethargy, and other signs of disease, such as lameness. All were from the same farrowing time to ensure uniform age and production category, with no significant differences in age or health status before assignment to experimental groups. As the piglets were vaccinated on day 21, the data collected on day 21 correspond to the antibody and PCR results before immunization. Clinical signs related to PCVAD and mortality were recorded until the pigs were sold to the slaughterhouse at age 26 weeks.

### 2.2. Serum Samples

This study included 44 piglets born to four sows that had been vaccinated with the Ingelvac CircoFLEX^®^ PCV2 vaccine (Boehringer Ingelheim Animal Helath Japan Inc., Tokyo, Japan). Each piglet was individually identified by an ear tag. The piglets were divided 1:1 into two groups, with 22 vaccinated with the Ingelvac CircoFLEX^®^ PCV2 vaccine at age 21 days and 22 remaining non-vaccinated. All piglets were kept under the same management conditions adjacent to the piggery on the farm. Overall, 100 blood samples were taken randomly from the vaccinated and non-vaccinated groups. To ensure a sample size of five for statistical analysis and to mitigate the impact of accidental deaths, samples were randomly collected from each group. Five blood samples each were collected from each group at 21, 28, 35, 41, 48, 63, 91, and 145 days of age, and 10 blood samples were collected from each group at the age of 173 days. All serum samples were stored at −80 °C.

### 2.3. PCV2 and PRRS Gene Detection by qPCR/qRT-PCR

Viral DNA and RNA were extracted from pig serum and virus isolation cells. Virus-infected cells were lysed by three rounds of freezing and thawing and centrifuged to obtain supernatants. DNA and RNA were extracted from 200 µL aliquots of these supernatants and pig serum samples with an automated extraction machine, MagLEAD^®^ 12gC (Precision System Science Co., Ltd., Matsudo, Japan), according to the manufacturer’s instructions. The extracted DNA and RNA were stored at −20 °C until use.

The PCV2 gene copy number in the DNA extracts was quantified using qPCR. Each 50 μL reaction contained 1 μL of each primer at 10 pmol/μL, 5 μL of 5 × PCR buffer, 2.5 μL of 25 mM MgCl_2_, 0.75 U of Taq DNA polymerase, 1 μL of 5 mM dNTP stock solution, 11.35 μL of DEPC-treated water, and 2.5 μL of extracted DNA. The conserved region of PCV2 genomes was targeted using the following primers: Circo-Gen-F (5′-GGCCACCTGGGTGTGGTAAA-3′) and Circo-Gen-R (5′-CCCACCACTTGTTTCTAGGTGGTT-3′), and the probe Circo-Gen-Probe (5′-6-FAM-TTTGCAGACCCGGAAACCACATACTGGA-BHQ-1-3′) [19]. The amplification protocol consisted of an initial denaturation at 94 °C for 5 min, followed by 40 cycles of denaturation at 95 °C for 30 s denaturation, annealing at 53 °C for 30 s, extension at 72 °C for 40 s, and a final elongation at 72 °C for 7 min. PRRSV gene copy number in the RNA extracts was quantified by qRT-PCR as described [19]. The gene copy number of PCV2/PRRSV was determined through quantitative qPCR/qRT-PCR using synthetic DNA/RNA standards with known copy numbers. These standards were serially diluted to create a standard curve, and the copy number in the samples was calculated by comparison with the curves.

### 2.4. Antibody Responses Induced by PCV2 and PRRSV

Enzyme-linked immunosorbent assays (ELISAs) were outsourced to a specialized testing company (Shokukanken Inc., Maebashi, Japan). Serum samples collected from pigs at ages 21, 63, 91, 145, and 173 days were submitted to the company, and specific IgG antibodies against PCV2 and PRRSV were measured with ELISAs. The PCV2 ELISA was developed in-house by Shokukanken Inc. and used a recombinant partial ORF2 protein of PCV2 expressed in Escherichia coli as the antigen. Optical density (OD) was measured at 450 nm. Antibody titers (units) were calculated based on a standard curve generated from serial dilutions of a reference serum provided by the company. IDEXX Laboratories, Inc. (Westbrook, ME, USA) provided the PRRSV ELISA kit.

### 2.5. PCV2 Isolation from Serum Samples

Serum samples were obtained from ten vaccinated and ten non-vaccinated pigs at age 173 days. PK-15 porcine kidney cells free of porcine circovirus 1 were kindly provided by Dr. Makoto Ozawa (Kagoshima University, Japan). These cell lines were maintained in high-glucose-containing Dulbecco’s modified Eagle’s medium (DMEM; Nacalai Tesque, Kyoto, Japan), supplemented with 10% heat-inactivated fetal bovine serum (FBS; Gibco, NY, USA) and 1% penicillin–streptomycin (Pe/St; Nacalai Tesque) at 37 °C under 5% CO_2_. Each serum sample was diluted to 10% in DMEM without FBS and inoculated onto PK-15 cells (2.0 × 10^4^ cells/mL, passage number 10) in three wells of a 24-well plate. After 90 min, the medium was replaced with DMEM containing 2% FBS, and the cells were cultured for about 1 week. The supernatants were collected, and the cells were lysed by three rounds of freezing and thawing. Each supernatant and cell lysate was inoculated onto new PK-15 cells, followed by three blind passages. The presence of PCV2 in each supernatant and cell lysate from the third blind passage was confirmed using qPCR and indirect immunofluorescent assays (IFAs).

### 2.6. Immunofluorescent Assays

Cell supernatants and lysates were inoculated onto PK-15 cells in 24-well plates for 5 days. The cells were fixed in 4% paraformaldehyde (Wako, Osaka, Japan), followed by incubation with primary mouse monoclonal anti-PCV2 capsid antibody (GT972) (ab308410; Abcam, Boston MA, USA) diluted 500-fold in 0.5% bovine serum albumin (BSA, Thermo Fisher Scientific Inc., Carlsbad, CA, USA) in PBS at 37 °C for 30 min. After washing with PBS, the cells were incubated with Alexa FluorTM Plus 488 goat anti-mouse IgG antibody (ab150113; Abcam) diluted 1000-fold in 0.5% BSA in PBS. The cells were examined under an EVOSTM AM7000 fluorescence microscope (Thermo Fisher).

### 2.7. Statistical and Correlation Analysis

All experiments were performed in triplicate. Differences between the vaccinated and not-vaccinated groups in mortality rates and qPCR PCV2 results were analyzed with a chi-square test. Between-group differences in ELISA results and mean viral loads were compared by two-way analysis of variance (ANOVA), followed by Tukey’s multiple comparisons test. Statistical analyses were performed using Microsoft Excel (Microsoft Corporation, Redmond, WA, USA) and Prism 9 (GraphPad Software Inc., San Diego, CA, USA) software, with *p* ≤ 0.05 indicating statistical significance.

## 3. Results

### 3.1. PCV2 Copy Number in Vaccinated and Non-Vaccinated Pigs’ Serum

**Figure 1:**
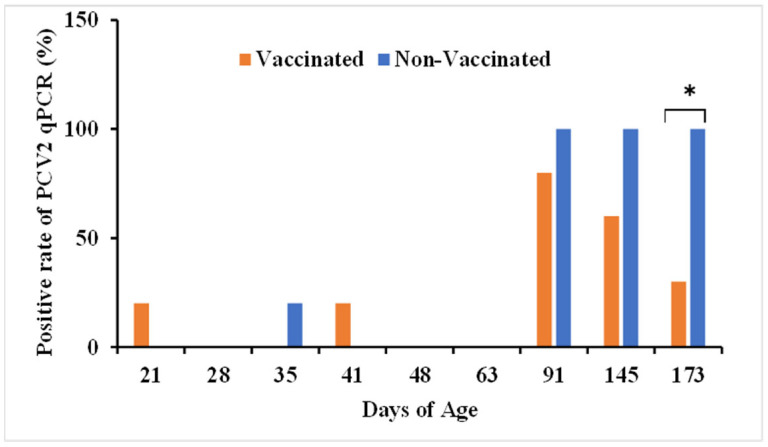
Percentages of PCV2-positive serum samples in vaccinated and non-vaccinated pigs over time, as determined with qPCR. Serum samples were obtained from five pigs in each group from day 21 to day 145 and from 10 pigs in each group on day 173. * *p* < 0.05 for between-group comparisons by chi-square or Fisher’s exact test. Each bar represents the mean of three independent experiments.

PCV2 DNA was quantified in serum samples of vaccinated and non-vaccinated pigs by comparing the positive rate of serum samples for PCV2 with qPCR and the number of viral genes. PCV2 DNA was first detected in vaccinated pigs at age 21 days and in non-vaccinated pigs at age 35 days (Figure 1). At ages 91 and 145 days, 80% (4/5) and 60% (3/5) of vaccinated pigs, respectively, tested positive for PCV2 DNA, compared with 100% (5/5) of non-vaccinated pigs, differences that were not statistically significant. At age 173 days, however, the PCV2-positivity rate was significantly lower in vaccinated (30%, 3/10) than in non-vaccinated (100%, 10/10) pigs (*p* < 0.05).

**Figure 2:**
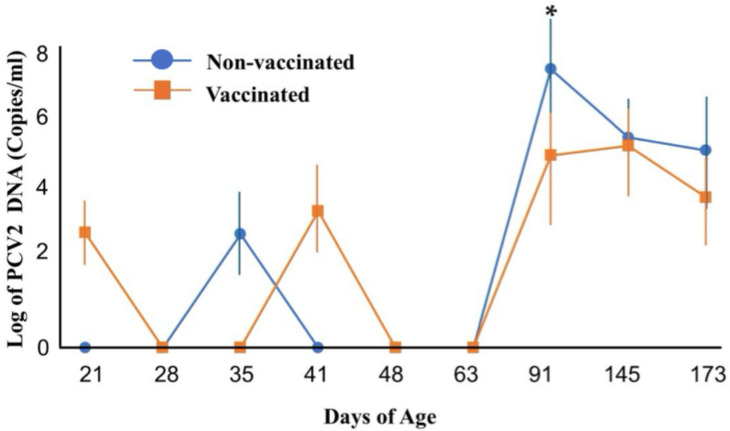
Mean PCV2 gene copy number in vaccinated and non-vaccinated pigs over time, as measured by qPCR. Error bars represent standard error. * *p* < 0.05 for between-group comparisons by two-way (ANOVA) followed by Tukey’s test. Each point represents the mean of three independent experiments.

Although the PCR-determined PCV2 positivity rate did not differ in the two groups at age 91 days, the mean PCV2 gene copy number was significantly lower in the vaccinated than in the non-vaccinated group (1.61 × 10^7^ copies/mL vs. 4.62 × 10^4^ copies/mL, *p* < 0.05) at this time point (Figure 2). Moreover, PCV2 viral gene copies remained lower in the vaccinated than in the non-vaccinated group through age 173 days.

### 3.2. Serological Test for PCV2 Antibody Detection

**Figure 3:**
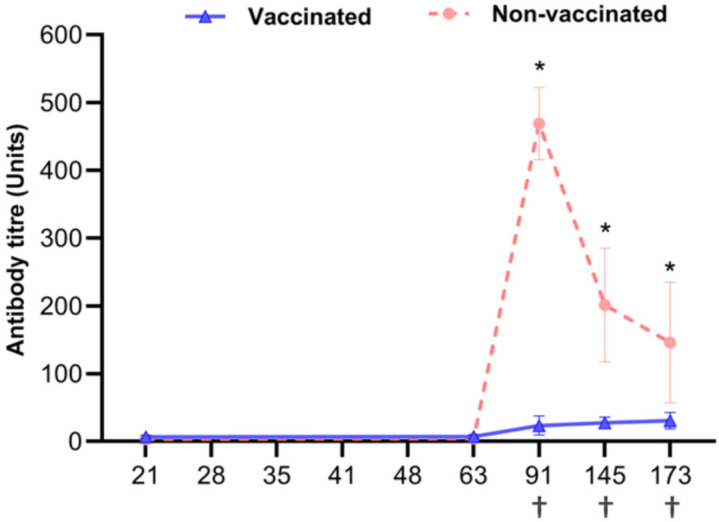
Mean anti-PCV2 antibody titers in vaccinated and non-vaccinated pigs over time, as measured using ELISAs. The Y-axis indicates unit values based on optical density (OD), measured at 450 nm. Antibody titers (units) were calculated based on a standard curve generated from serial dilutions of a reference serum provided by the company. The post-weaning mortality rate was significantly higher in the non-vaccinated group than in the vaccinated group (18.2% vs. 0%, *p* = 0.036). PCV2 antibody titers at 91, 145, and 173 days of age were significantly lower in the vaccinated than in the non-vaccinated group (*p* < 0.0001). †: Accidental death case of piglets in the non-vaccinated group. * *p* < 0.0001 for between-group comparisons. Each point represents the mean of three independent experiments.

Serological assays showed that all serum samples were positive for anti-PCV2 antibodies at age 21 days, with mean titers in the vaccinated and non-vaccinated groups of 3.0 ± 308 and 3.02 ± 368 ELISA units, respectively (Figure 3). A result of 3 or more units is considered positive. Although all results were above 3 units and thus positive, the higher antibody levels in the non-vaccinated group may give the impression that the antibodies did not increase in the vaccinated group. On day 91, anti-PCV2 antibody titers were increased significantly in both groups but were significantly lower (*p* < 0.0001) in the vaccinated than in the non-vaccinated group at 91, 145, and 173 days of age.

### 3.3. Clinical Signs and Mortality

No clinical signs related to PCVAD were observed during the study in the vaccinated group; however, four piglets in the non-vaccinated group were found dead at 70, 100, 120, and 125 days of age (Figure 3). The spleens of the deceased pigs were found to have a high PCV2 gene copy number. The post-weaning mortality rate was significantly higher in the non-vaccinated group (18.2%, 4/22) than in the vaccinated group (0%, 0/22) (*p* = 0.036).

### 3.4. PCV2 Isolation

**Figure 4:**
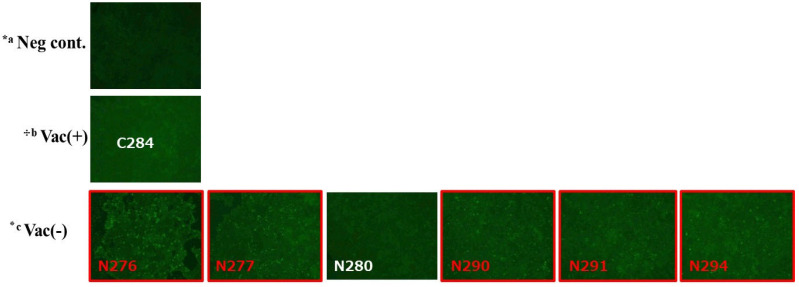
Confirmation of intracellular PCV2 antigen in PCR-positive serum samples from vaccinated and non-vaccinated pigs using an IFA. Red boxes and red letters show positive results, with the letters *a, ^⸭^b, and ٭c indicating negative controls, vaccinated, and non-vaccinated pigs, respectively. The results shown are from three independent experiments.

Attempts were made to isolate PCV2 from thirteen serum samples that tested PCR-positive for PCV2 at the age of 173 days, three from the vaccinated group and ten from the non-vaccinated group (Table 1). The infectivity of isolated PCV2 in PK-15 cells was confirmed using an IFA (Table 1, Figure 4). PCV2 was successfully isolated from five samples in the non-vaccinated group (50%) but from none in the vaccinated group (0%).

### 3.5. PRRSV Infection-Based Antibody Test and RT-PCR

**Figure 5:**
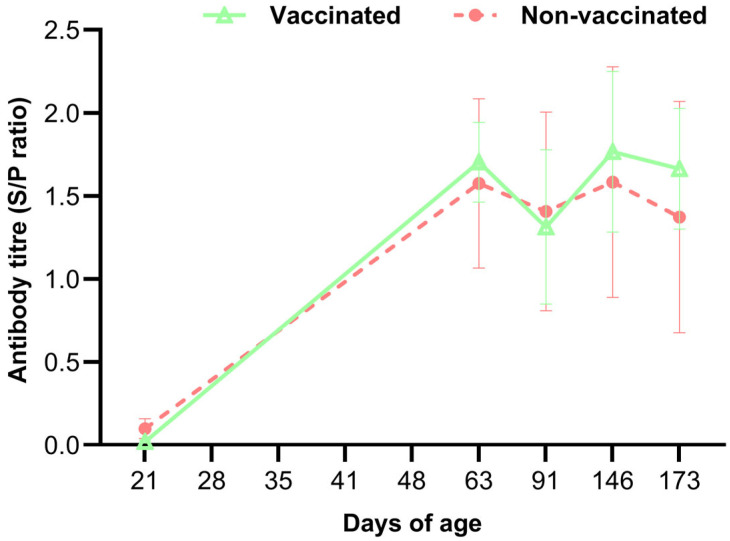
Mean anti-PRRSV antibody titers in vaccinated and non-vaccinated pigs over time, as measured using an ELISA. Each point represents the mean of three independent experiments.

Antibodies against PRRSV increased at 63 days in both the vaccinated and non-vaccinated groups, although their titers remained very low (Figure 5). None of these pigs had any clinical symptoms, and their titers remained constant until day 173. Mean anti-PRRSV antibody titers did not differ significantly in the vaccinated and non-vaccinated groups. All qRT-PCR tests for PRRSV were negative.

## 4. Discussion

The present study showed that vaccination against PCV2 reduced the incidence of accidental deaths due to PCVAD. Although PCV2 gene copy numbers differed significantly in the vaccinated and non-vaccinated groups at age 91 days (*p* < 0.05), overall PCR positivity rates and gene copy numbers did not differ significantly in these two groups. Virus isolation rates differed significantly in these two groups, and the induction of anti-PRRSV antibodies occurred shortly before PCV2 infection at 91 days of age.

Although qPCR positivity rates did not differ significantly in the vaccinated and non-vaccinated groups, significant differences in PCV2 gene copy number and anti-PCV2 antibody titers were observed. These results suggest that PCV2 replication occurred in both groups at age 91 days but that the vaccine effectively inhibited viral replication at that time. Therefore, it can be inferred that anti-PCV2 antibody induction levels in the vaccinated group were significantly lower compared to the non-vaccinated group. Furthermore, no infectious virus was detected in the vaccinated group, and there were no fatalities due to PCVAD.

The detection of the PCV2 gene using qPCR in the vaccinated group was not associated with PCV2 viremia or clinical symptoms. The results of PCV2 isolation suggest, therefore, that qPCR for PCV2 was unable to distinguish between live viruses and viral gene fragments in the serum samples. Additionally, the immune response by vaccination may have weakened the virus, causing fragmented viral genetic material to remain in the system. qPCR might have detected this fragmented DNA, even though viral replication was lower in the vaccinated group. PCV2 viremia levels and mortality rates were lower in the vaccinated group, indicating that, despite the qPCR results, the vaccine had sufficient efficacy. Lower levels of viremia have been found to contribute to reduced shedding of the PCV2 virus in pig farms [4,20] and reduced mortality from conditions such as PCVAD [15,21]. Furthermore, PCV2 gene copy numbers did not significantly affect average daily weight gain [7]. The present study found no between-group difference in the overall PCR positivity rate, suggesting that PCR testing alone may not be sufficient for evaluating the effectiveness of vaccination. Moreover, qPCR alone may not be sufficient to assess PCV2 control [9,22].

Although both clinically and serologically at a very low level, a weak immune response to PRRSV was observed on day 63 in both vaccinated and non-vaccinated pigs, with an immune response to PRRSV occurring prior to PCV2 infection at 91 days. These findings suggest that the immune response to PRRSV may be involved in PCV2 replication in pigs. Indeed, PRRS infection was reported to predispose pigs to PCV2 replication. [18,21,23,24]. PRRSV has been found to enhance PCV2 replication and associated lesions [20,23,25]. Furthermore, PRRSV was found to induce PCV2 replication in vitro [26]. It has been proposed that PRRSV may have an immunomodulating effect on pigs, leading to enhanced replication of PCV2. The poor induction of type I IFN response during PRRSV infection suggests that PRRSV may have adopted various strategies to suppress the induction and function of IFNs-α/β. [27]. Subsequent studies showed that suppressor of cytokine signaling 1, a crucial intracellular negative regulator of innate immunity, can be co-opted by PRRSV to evade host immune responses, thereby facilitating viral replication. [26]. Additionally, it has been reported that PRRSV proteins inhibit the IFN signaling pathway. [27,28]. These immune suppression mechanisms of PRRSV may be enhancing the replication of PCV2. Increased PCV2 viremia due to co-infection may, therefore, increase the risk of developing PCVAD. [24]. The results of the present study suggest that PCV2 vaccination may protect against PCVAD caused by PCV2 replication following co-infection with PRRSV.

## 5. Conclusions

This study aimed to evaluate the efficacy of a PCV2 vaccine in piglets on a farm using qPCR and virus isolation. In addition, the mechanism by which PRRSV co-infection or sequential infection affects PCV2 status was investigated. This study found that vaccination against PCV2 efficiently reduced the negative effects of PCV2 replication and effectively controlled PCV2 infection under field conditions. However, qPCR detection of PCV2 genes in blood was not sufficient to assess the effectiveness of the vaccine. Rather, the introduction of other testing methods, such as virus isolation, was necessary. Furthermore, sudden changes in PCV2 viral load may be due to co-infection with another virus, such as PRRSV. Results of the study further suggest that PCV2 vaccination may protect against PCVAD caused by PCV2 replication following co-infection with PRRSV, and the vaccine reduced PCVAD damage.

## Figures and Tables

**Table 1:** PCV2 gene copy numbers in serum and cell supernatant, and the results of PCV2 isolation confirmed with an IFA.

ID	Serum Copies/mL	Supernatants Copies/mL	IFA +/−
C281	5.00 × 10^3^	<LOD	ND
C284	5.01 × 10^2^	9.00 × 10^1^	−
C292	3.10 × 10^3^	<LOD	ND
N276	1.78 × 10^4^	3.52 × 10^2^	+
N277	1.94 × 10^4^	1.74 × 10^2^	+
N280	1.05 × 10^4^	9.87 × 10^1^	−
N282	2.73 × 10^3^	<LOD	ND
N283	1.22 × 10^3^	<LOD	ND
N284	9.15 × 10^2^	<LOD	ND
N290	4.65 × 10^5^	8.94 × 10^1^	+
N291	1.17 × 10^5^	1.83 × 10^2^	+
N294	1.42 × 10^4^	9.73 × 10^1^	+
N297	3.25 × 10^3^	<LOD	ND

<LOD; below the limit of detection, ND; not done.

## Data Availability

The data presented in this study are available from the corresponding author upon reasonable request.

## Abbreviations

The following abbreviations are used in this manuscript:

PCV2	Porcine circovirus type 2
PRRSV	Porcine reproduction and respiratory syndrome virus
ELISA	Enzyme-linked immunosorbent assay
